# Bergamottin (Ber) ameliorates the progression of osteoarthritis via the Sirt1/NF-κB pathway

**DOI:** 10.3389/fphar.2024.1389786

**Published:** 2024-04-29

**Authors:** Guangjie Shen, Weihao Zhang, Qiming Tu, Juncheng Wang

**Affiliations:** Department of Orthopaedic Surgery, The Third Hospital Affiliated to Wenzhou Medical University, Rui’an, China

**Keywords:** osteoarthritis, Sirt1/NF-κB, Bergamottin, chondrocytes, anti-inflammation

## Abstract

Osteoarthritis (OA) is a common chronic disease characterized by progressive cartilage degeneration and secondary synovial inflammation. Bergamottin (Ber) is an important natural derivative of the furanocoumarin compound, extracted from natural foods, such as the pulp of grapefruits and pomelos. Ber exhibits several characteristicsthat are beneficial to human health, such as anti-inflammation, antioxidant, and anti-cancer effects. However, the role of Ber in the treatment of OA has not been elucidated to date. Therefore, in the present study, *in vitro* experiments were conducted, which demonstrated that Ber reduces the secretion of inducible nitric oxide synthase (iNOS), nitric oxide (NO), cyclooxygenase-2 (COX2), tumor necrosis factor-α (TNF-α), interleukin-6 (IL-6) and prostaglandin E2 (PGE2) under the stimulation of interleukin-1β (IL-1β). Ber also reversed the IL-1 β-mediated aggrecan and type II collagen degradation within the extracellular matrix (ECM). In addition, *in vivo* experiments were conducted, in which Ber ameliorated the progression of OA in mice. It was revealed that Ber exerted its cellular effect by activating the Sirt1/NF-kB pathways. In conclusion, the present study demonstrated the therapeutic potential of Ber in the context of OA.

## 1 Introduction

Osteoarthritis (OA) is a common bone disease, which is generally accompanied by the loss of articular cartilage, fibrosis, and damage to the entire articular surface (Pereira et al., 2015; Abramoff and Caldera, 2020). With the increase in human life expectancy, both the incidence and prevalence of osteoarthritis are on a rapid rise (Hunter et al., 2014). The main symptoms of OA include swelling around the affected joints, stiffness, reduced range of motion at the joints, joint pain, and limited mobility. OA is more prevalent among middle-aged and elderly individuals, and females gender is affected (Loeser, 2009). The traditional treatment of OA includes pain management and articular prosthesis for end-stage diseases. While the specific pathogenesis of OA remains to be elucidated to date, several factors reportedly influence the progression of osteoarthritis, including obesity, aging, trauma, inflammation, osteoporosis, and joint deformities (Millerand et al., 2019; Katz et al., 2021). Among these factors, inflammation is considered to play a crucial role in the occurrence and development of osteoarthritis (Arra and Abu-Amer, 2023). Interleukin-1β (IL-1β) is one of several inflammatory factors that can lead to the occurrence of osteoarthritis by inducing the overexpression of related metabolic enzymes and inflammatory cytokines, such as NO, PGE2, ADAMTS, and MMPs (Lin et al., 2020a). This ultimately resulting in the degradation of the extracellular matrix. The joint cartilage tissue comprises type II collagen, glycosaminoglycans, and chondroitin sulfate, all of which together form the extracellular matrix (Lin et al., 2020b). Inflammatory factors mediate the production of a large number of several metabolic enzymes and inflammatory cytokines, it ultimately leading to the degradation of the extracellular matrix and resulting in the occurrence of osteoarthritis (Theeuwes et al., 2021).

Several studies have reported a significant increase in the expression of IL-1β in the serum and synovial fluid of osteoarthritis patients (Au et al., 2007; Santangelo et al., 2012). Therefore, inhibiting the expression of IL-1β could be used as a potential target in the treatment of osteoarthritis.

NF-κB is a highly classical molecular regulatory pathway that can promotes the occurrence and development of inflammation (Barnes and Karin, 1997). The release of various pro-inflammatory factors facilitates the expression and nuclear translocation of nuclear factor-kappa B (NF-κB) can be facilitated (Kumar et al., 2004). The activation of NF-κB then induces expressions of MMPs and the degradation of the cartilage matrix. Silent Information Regulator Factor 1 (SIRT1) is a member of the mammalian sirtuin family of proteins. It is a conserved nicotinamide adenine dinucleotide (NAD)+-dependent deacetylase (Xie et al., 2013). It plays a regulatory role in various biological processes, including cellular metabolism, gene silencing, cell survival, and DNA repair. SIRT1 is a homolog of Sir2 in mammals, the latter belonging to the class III of histone deacetylases (Shah et al., 2017). SIRT1 reportedly deacetylates various non-histone substrates and histones. Research has demonstrated that SIRT1 could reduce the expressions of inflammatory factors by inhibiting NF-κB (Jia et al., 2022). Therefore, the SIRT1/NF-κB pathway could be an important signaling pathway involved in the regulation of both the normal development and pathological destruction of cartilage.

Current therapeutic approaches are limited to the use of hyaluronic acid, opioids or corticosteroids. However, these only relieve articular pain and swelling, with overuse leading to deleterious side effects. Therefore, an effective and safe drug that relieves clinical symptoms of OA while also decelerating its progression has to be developed. Ber is a natural furanocoumarin compound with anti-inflammatory, antioxidant, and anti-aging properties (Wang et al., 2022). Studies have revealed that Ber inhibits the invasive activity of human glioma cells’ matrix metalloproteinase 9 in human glioma cells by regulating the activity of Rac1(Hwang et al., 2010). Additionally, Ko et al. reported Ber can suppressed the epithelial-mesenchymal transition and blocks the downregulation of fibronectin through the PI3K/AKT/mTOR pathway (Ko et al., 2018). Research by Kim et al. suggests that Ber can inhibit the NF-κB pathway, thereby exerting anti-cancer effects (Kim et al., 2016). While the above findings suggest that Ber exhibits significant anti-inflammatory therapeutic effects, its role in osteoarthritis has not been reported so far.

Therefore, the present study aimed to elucidate the role of Ber on the anti-inflammatory activity and ECM of chondrocytes and cartilage in OA. The study involved the use of *in vitro* and *in vivo* experimental models to investigate the anti-inflammatory properties of Ber and unravel the mechanisms underlying the effects of Ber on the pathogenesis of OA.

## 2 Materials and methods

### 2.1 Reagents

Bergamottin (Ber) (purity >98%) and Phosphate buffered saline (PBS) was obtained from ChemeGen BioTechnology (Shanghai, China). Anti-Lamin B1 and anti-IκB were acquired from Proteintech (Wuhan, China). Anti-iNOS, anti-Sirt1, anti-P65, anti-GAPDH, Anti-Sirt1, and anti-COX-2 were purchased from Bioss (Beijing, China). EX-527 and MTT were purchased from Sigma-Aldrich (CA, USA).

### 2.2 Culture of the primary chondrocytes

The chondrocyte extraction and culture were performed according to the previous studies (Xian et al., 2022). Chondrocytes were isolated from the 5-day-old mice. Knee joint cartilage was exposed under sterile conditions and collected into a PBS solution. Then, treat cartilage tissue with type II collagenase. Finally, chondrocytes were cultured in DMEM/F12 medium (10% FBS and 1% penicillin/streptomycin) in an incubator with 5% CO_2_ at 37°C.

### 2.3 Animal studies

C57BL/6 mice were obtained from Wenzhou Medical University. The model of OA was established by meniscus injury surgery. Mice were randomly separated into three groups (*n* = 6 in each): a control group (sham-operated), OA group (OA), and OA treated with Ber group (Ber). Mice were intraperitoneally injected with Ber (20 mg/kg) once a day for eight consecutive weeks. The mice were reared in specific pathogen-free cages, given unrestricted movement, and provided autoclaved food and water. All animal experiments were carried out and approved by the Animal Administration Committee of Wenzhou Medical University.

### 2.4 Cell viability assays

The Cell counting Kit 8 (CCK-8) reagent is a commonly used method for measuring cell viability. The chondrocytes were seeded into 96-well plates and treated with concentrations of IL-1β (10 ng/mL) and Ber (0–40 μM) for 24 or 48 h, respectively. The effect of Ber on cells was measured by using a spectrophotometer (Synergy HT, Bio-Tek, United States).

### 2.5 ELISA

Chondrocytes (3 × 10^5^ cells per mL) were seeded in 6-well plates and treated with Ber(5, 10, or 20 μM) 24 h prior to the addition of IL-1β (10 ng mL^−1^). They were incubated for 24 h and then the concentration of NO was measured using the Griess reaction as previously described (Au et al., 2007). The optical density of each sample was measured at a wavelength of 543 nm. The culture medium supernatant from each sample was collected, the levels of PGE2, Aggrecan, ADAMTS-5, and IL-6 in the culture medium were measured with an ELISA kit (R&D Systems, Minneapolis, MN, United States) according to the manufacturer’s instructions.

### 2.6 Western blotting

The cell proteins were isolated using RIPA lysis. The concentration of proteins was measured by the BCA Assay Kit. Protein samples (20 μM) were separated by SDS-PAGE electrophoresis and electrotransferred to the PVDF membrane (Millipore, MA, United States). The protein sample was transferred to a membrane and sealed in 5% nonfat milk for 1 h. The primary antibody (1:1000) was incubated overnight at 4°C. After being washed with TBST, the membranes were incubated with secondary antibodies for 60 min. Odyssey Infrared Imaging System (LI-COR, United States) was used to obtain images. The blot images were detected by SuperSignal West Pico PLUS Chemiluminescent Substrate (Thermo Fisher)

### 2.7 Molecular modeling

First, we mapped the molecular structure of SA using ChemBioDraw and minimized its energy using ChemBio3D. Based on the requirements of current molecular docking experiments, we downloaded the corresponding Sirt1 from the PDB website (https://www.rcsb.org/). After being processed in PyMoL (version 1.7.6), the lowest energy conformations for docking were decided based on default parameters. When the molecular structures of Ber and Sirt1 required for docking analysis were finalized, subsequent molecular docking analysis was performed by AutoDockTools (version 1.5.6). The final 3D views of the images were obtained via PyMoL.

### 2.8 Immunofluorescence staining

The chondrocytes were seeded into 6-well plates and treated with Ber(20 μM). Then chondrocytes were fixed with 4% paraformaldehyde (PFA, Servicebio, Wuhan, China) for 10 min and permeabilized with 0.1% Triton X-100 (BioFroxx, Germany) for 15 min. The sections were blocked with 5% bovine serum albumin. The samples were incubated with the primary antibody overnight at 4°C, followed by incubation with the fluorescent secondary antibody for 60 min. The next day, samples were treated with secondary antibodies for 45 min and stained with DAPI for 15 min. Finally, the samples were examined using an inverted fluorescence microscope (Leica DMIL, Germany).

### 2.9 Histopathologic analysis

The cartilage tissues of mice were fixed with 10% paraformaldehyde, then dehydrated, embedded, sectioned, and stained with hematoxylin-Eosin and Safranin O/Fast Green staining. The bright-field images were captured using a microscope (Thermo Fisher) and processed with ImageJ software (v1.8.0).

### 2.10 Statistical analysis

GraphPad Prism 7.0 software was used to execute statistical analyses and create the statistical charts. One-way ANOVA followed by Tukey’s t-tests multiple comparisons was used to compare the groups. *p* < 0.05 indicates a statistically significant difference.

## 3 Results

### 3.1 Potential viability of chondrocytes in the presence of Ber

**FIGURE 1:**
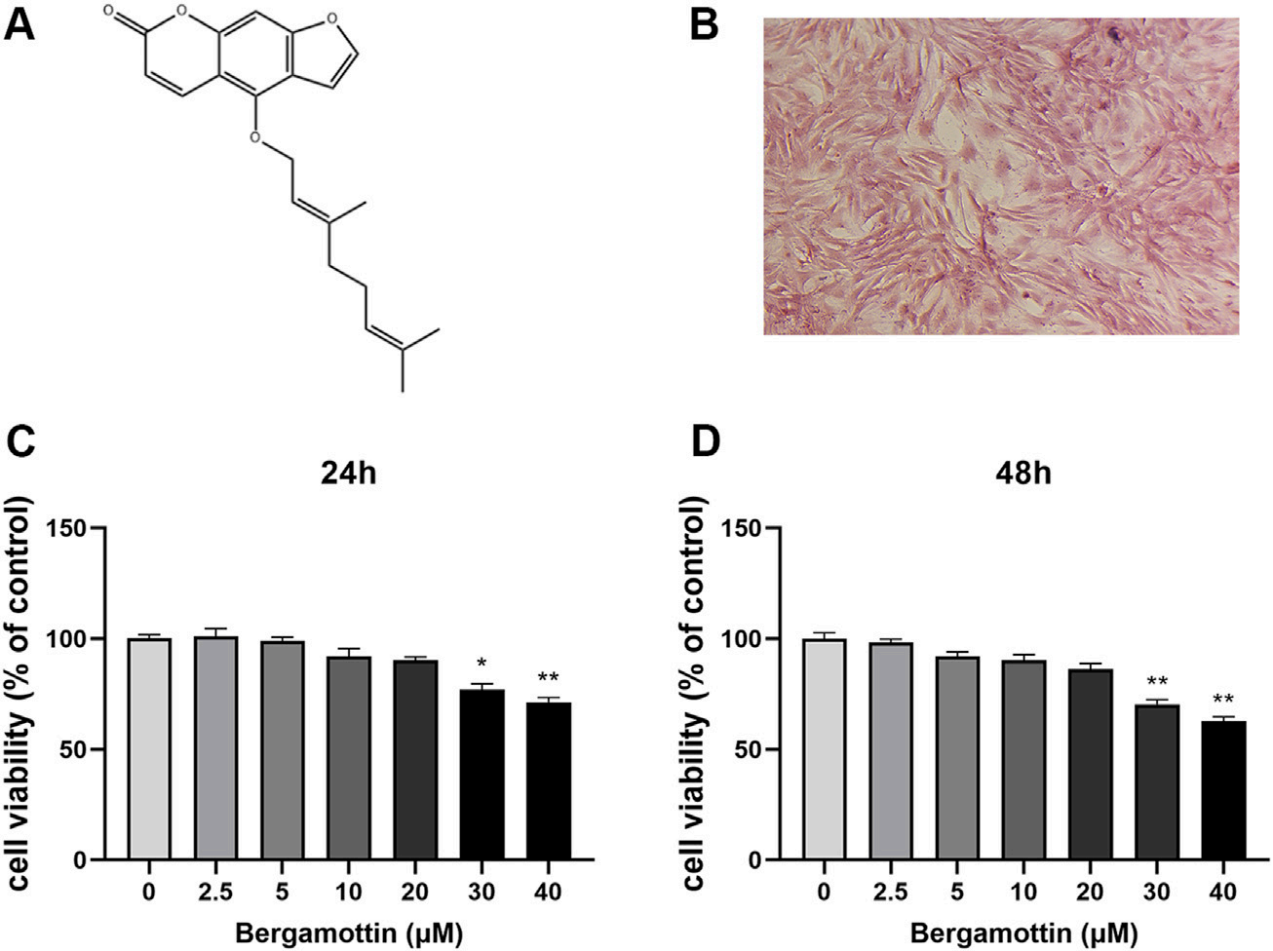
Cytotoxic analysis of Ber. **(A)** Chemical structure of SA. **(B)** The Safranin O staining of chondrocytes. **(C, D)** Cells were cultured with different concentrations of Ber (0, 2.5, 5, 10, 20, 30, and 40 μM) for 24 h and 48 h. The cell viability was determined by CCK-8 assay. *p* < 0.05 vs. control group; **p < 0.01 vs. control group, *n* = 5.

Figure 1A illustrates the molecular structure of Ber. The Safranin O staining results for cellular proteoglycans are presented in Figure 1B. The cytotoxic effect of Ber on chondrocytes was assessed using the CCK-8 assay. First, the chondrocytes were treated with different concentrations of Ber (0, 2.5, 5, 10, 20, 30, and 40 μM) for 24 and 48 h. Subsequently, the toxicity of Ber was assessed using the CCK-8 reagent. As presented in the results in Figures 1C,D, Ber exerted no toxic effects on chondrocytes within the concentration range of 5–20 μM, while the Ber concentrations 30 μM resulted in evident cytotoxicity. Therefore, Ber concentrations of 5, 10, and 20 μM were selected for the subsequent cell experiments to be conducted in the present study.

### 3.2 Effects of Ber on IL-1β-induced inflammation in chondrocytes

**FIGURE 2:**
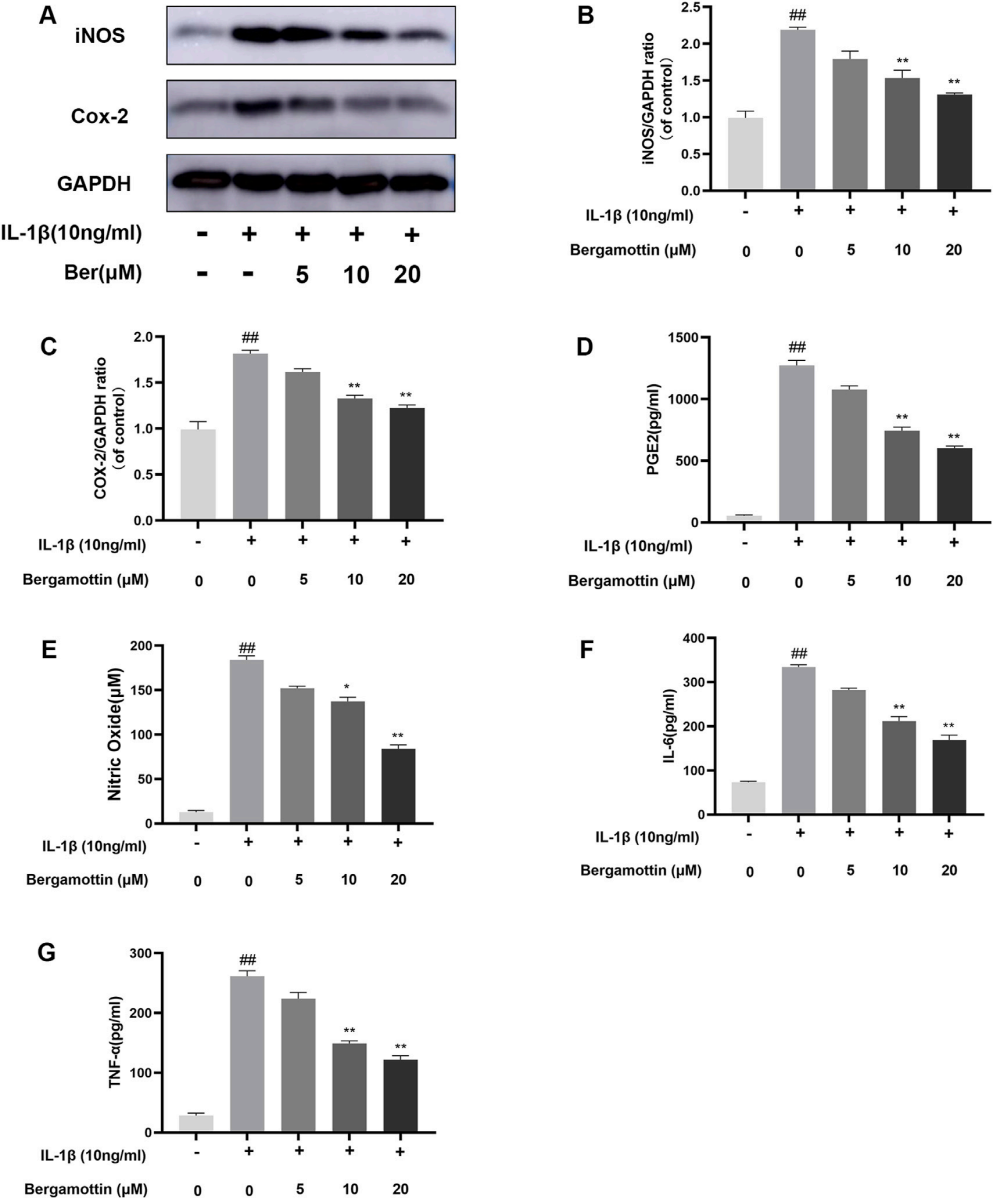
Ber inhibits IL-1β-induced PGE2, NO, IL-6, TNF-α, COX-2 and iNOS production. **(A–C)**: The level of INOS and COX-2 in the chondrocytes. **(D–G)**: The levels of NO, PGE2, and IL-6 in the chondrocytes were measured by ELISA. ##*p* ≤ 0.01 vs. control group; **p < 0.01 vs. IL-1β group, *n* = 5.

IL-1β induction leads to the production of various inflammatory cytokines in chondrocytes, ultimately causing inflammation in these cells. Whether Ber could inhibit IL-1β-induced inflammation in chondrocytes was assessed in the present study. ELISA and Western blotting were performed to detect the expression levels of various inflammatory cytokines, and the results are presented in Figures 2A–C. In comparison to the control group, the IL-1β group presented significantly increased expression levels of COX-2 and iNOS, while the Ber-treatment group exhibited reduced expression levels of IL-1β-induced COX-2 and iNOS in a dose-dependent manner. The ELISA results revealed a significant upregulation in IL-6, PGE2, TNF-α, and NO expression levels in the IL-1β-induced group. In contrast, the Ber group exhibited an inhibitory effect on the expression levels of IL-1β-induced NO, TNF-α, IL-6, and PGE2 (Figures 2D–G). The above results indicated that Ber could significantly inhibit IL-1β-induced inflammation in chondrocytes.

### 3.3 The effect of Ber on IL-1β-induced degradation of extracellular matrix in chondrocytes

**FIGURE 3:**
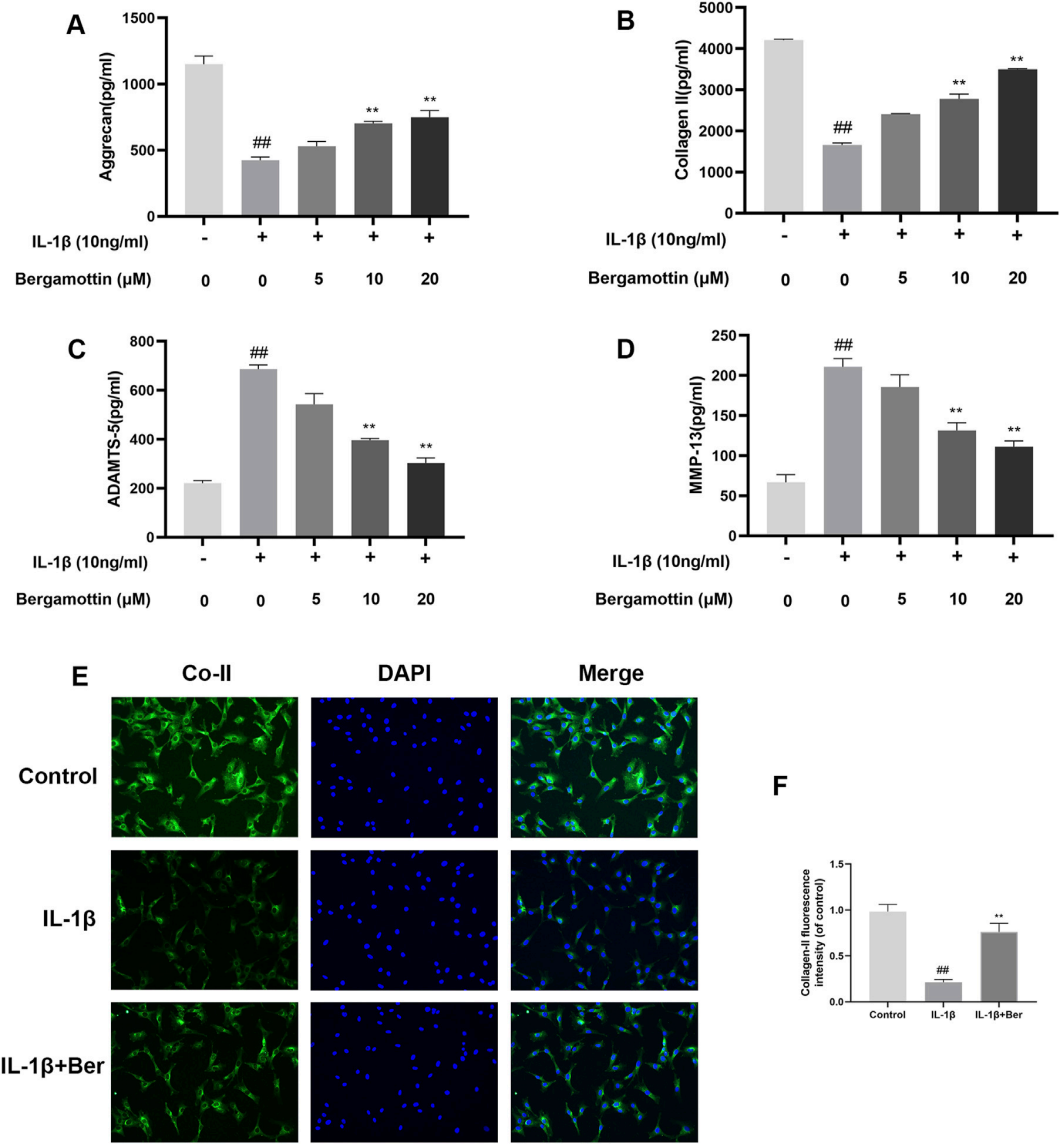
Ber inhibits ECM degradation of chondrocytes. **(A–D)**: The level of ADAMTS5 and Collagen II in the chondrocytes was evaluated by ELISA. **(E, F)**: Immunofluorescence staining for Collagen II and the nucleus was performed. P##*p* ≤ 0.01 vs. control group; **p < 0.01 vs. IL-1β group, *n* = 5.

Type II collagen and proteoglycans are the major components within chondrocytes, and the expressions of these reflect the functionality of chondrocytes. IL-1β induction leads to the production of metalloproteinases, which could lead to in the degradation of the extracellular matrix of chondrocytes. Therefore, the impact of Ber on IL-1β-induced degradation of the extracellular matrix of chondrocytes, was investigated in the present study using Western blotting to determine the expressions of metalloproteinases. The results are presented in Figures 3A–D, IL-1β induction led to abnormal expressions of MMP-13 and ADAMTS-5, while Ber significantly inhibited their expressions of these metalloproteinases. Collagen II fluorescence staining results revealed that Ber effectively inhibited the IL-1β-induced degradation. These findings suggested that Ber could significantly inhibit the IL-1β-induced degradation of the extracellular matrix (Figures 3E,F).

### 3.4 Effect of Ber on NF-κB in IL-1β-induced chondrocytes

**FIGURE 4:**
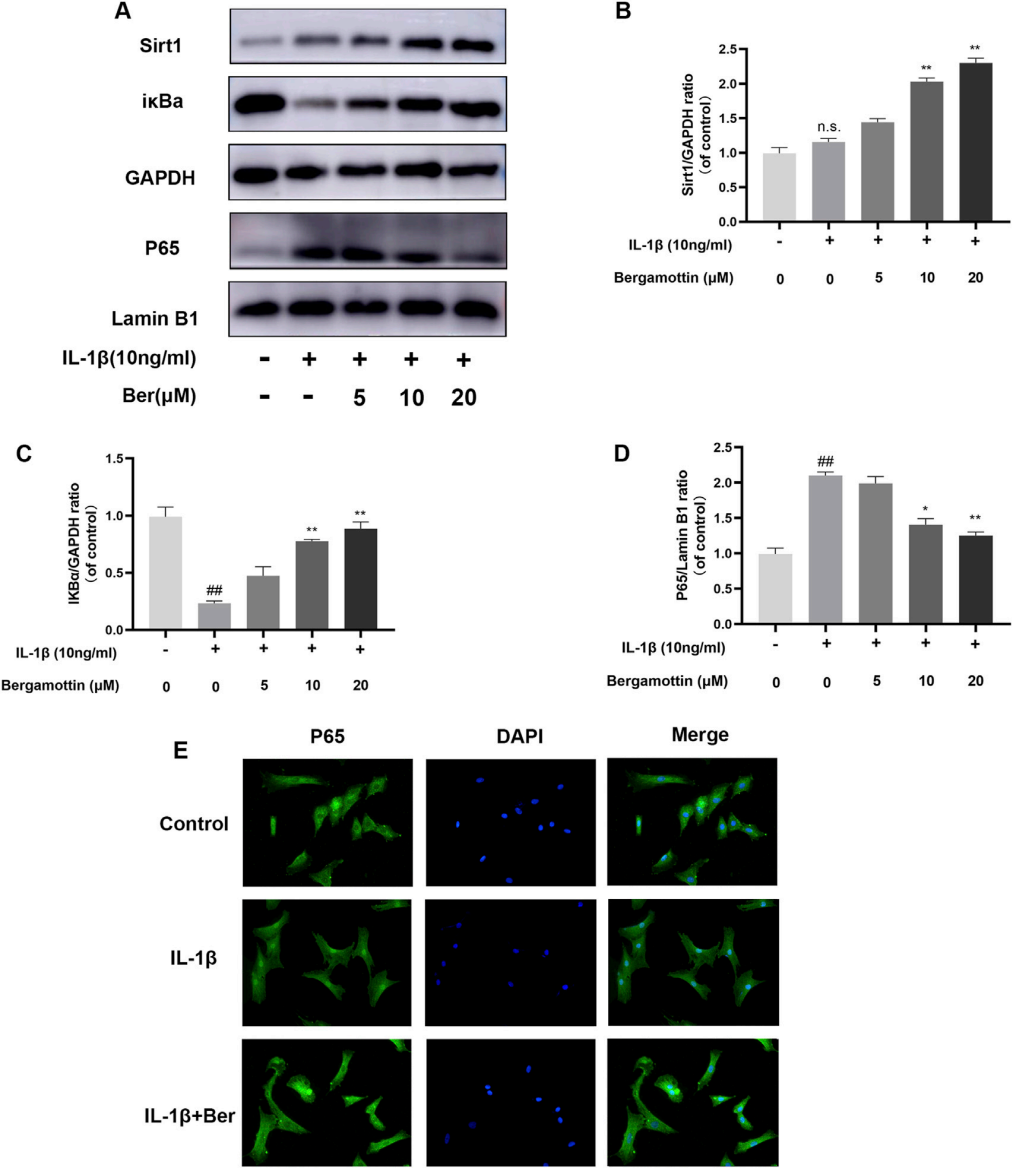
Ber inhibits IL-1β-induced NF-κB activation. **(** **A–D** **)**: The production of P65 and IκBα in chondrocytes. **(** **E** **)**: The p65 was observed by immunofluorescence. ##*p* ≤ 0.01 vs. control group; **p < 0.01 vs. IL-1β group, *n* = 5.

NF-kB plays a significant regulatory role in inflammatory diseases, and its activation could further exacerbate inflammation. Therefore, the impact of Ber on NF-kB in IL-1β-induced chondrocytes was investigated in the present study. Western bloting was conducted to evaluate the expressions of IκBα and P65, and the results are presented in Figures 4A–D. It was observed that under normal conditions, P65 expression was low, and IκBα expression was high. IL-1β then induced an increase in P65 expression, and Ber inhibited the expression of P65 in a dose-dependent manner. The chondrocytes were subjected to p65 cellular immunofluorescence staining.The results revealed that IL-1β could cause P65 to translocate to the nucleus of the cell., and Ber could inhibit this phenomenon (Figure 4E). The above results suggested that Ber inhibited the activation of NF-kB within chondrocytes, thereby exerting an inhibitory effect on IL-1β-induced inflammation in chondrocytes.

### 3.5 Ber inhibited NF-κB activation through the activation of Sirt1

**FIGURE 5:**
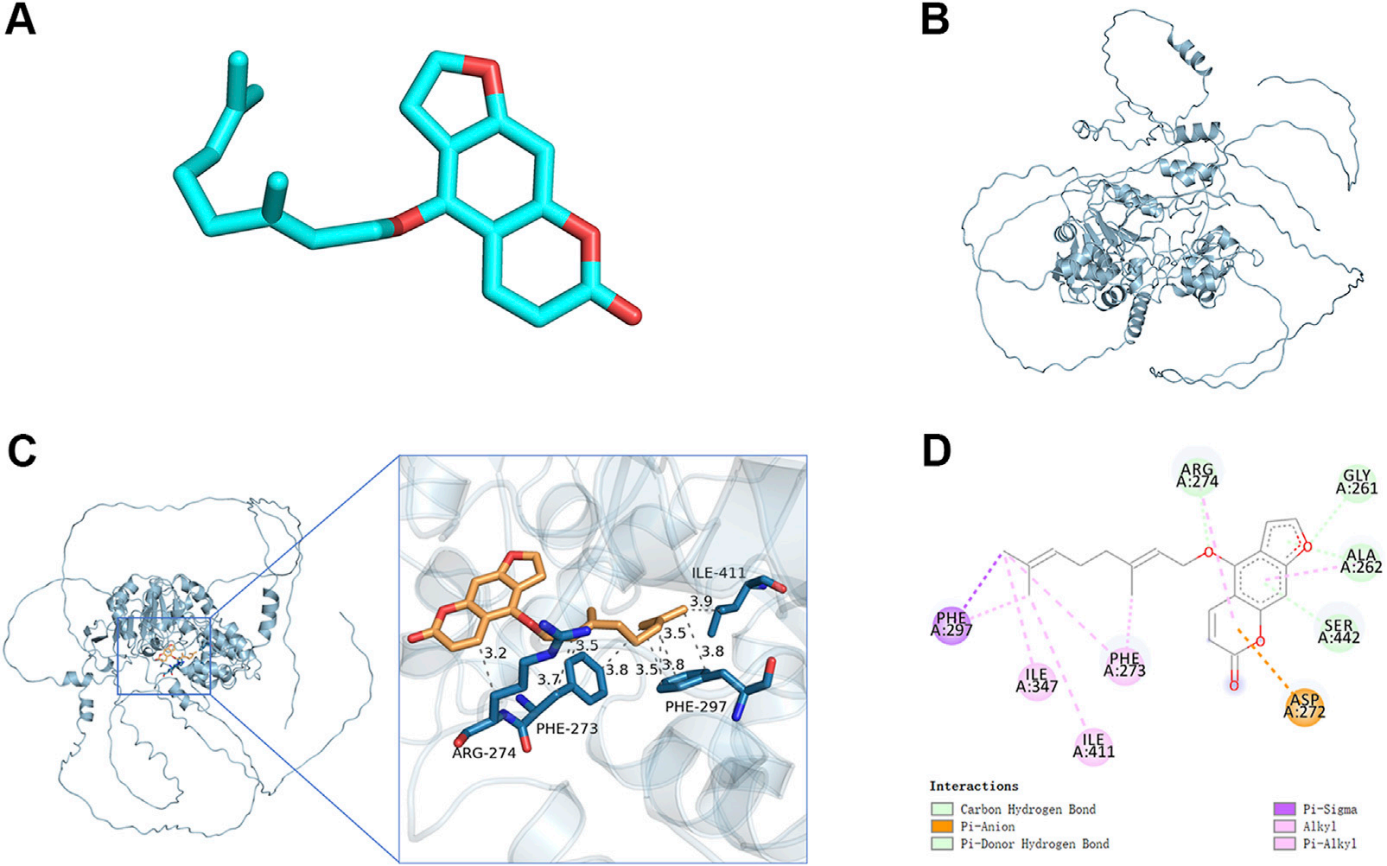
Molecular docking study of Ber to Sirt1. **(A)** 2D structure of Ber. **(B)** 2D structure of Sirt1. **(C, D)** The general view of Ber in the domain of Sirt1 structure according to the Space-filling model and Ribbon model. ##*p* ≤ 0.01 vs. control group; **p < 0.01 vs. IL-1β group, *n* = 5.

Sirt1 effectively inhibits the activation of NF-kB. In the molecular docking analyses conducted in the present study, Ber could bind to Sirt1 (Figures 5A–D). The small molecule Ber could bind to the SIRT1 protein through hydrophobic interactions with three residues: PHE273 at the distances of 3.5Å, 3.7Å, and 3.8Å; ARG274 at the distances of 3.2Å and 3.9Å; and ILE411 at a distance of 3.9Å. In addition, Ber exhibited hydrophobic interactions with four residues: PHE297 at distances of 3.5Å, 3.5Å, 3.8Å, and 3.8Å. Accordingly, it was inferred that presence of these interactions allowed the small molecule Ber to stably bind to the receptor protein SIRT1. Subsequently, the impact of Ber on IL-1β-induced Sirt1 in chondrocytes was investigated by assessing the expression levels of Sirt1 using Western blotting. Ber was observed to increase the expression levels of Sirt1 in a dose-dependent manner (Figures 4A,B). This finding suggested that the inhibition of IL-1β-induced NF-kB activation caused by Ber could have occurred through the activation of Sirt1.

### 3.6 EX-527 reversed the effect of Ber on IL-1β-treated chondrocytes

**FIGURE 6:**
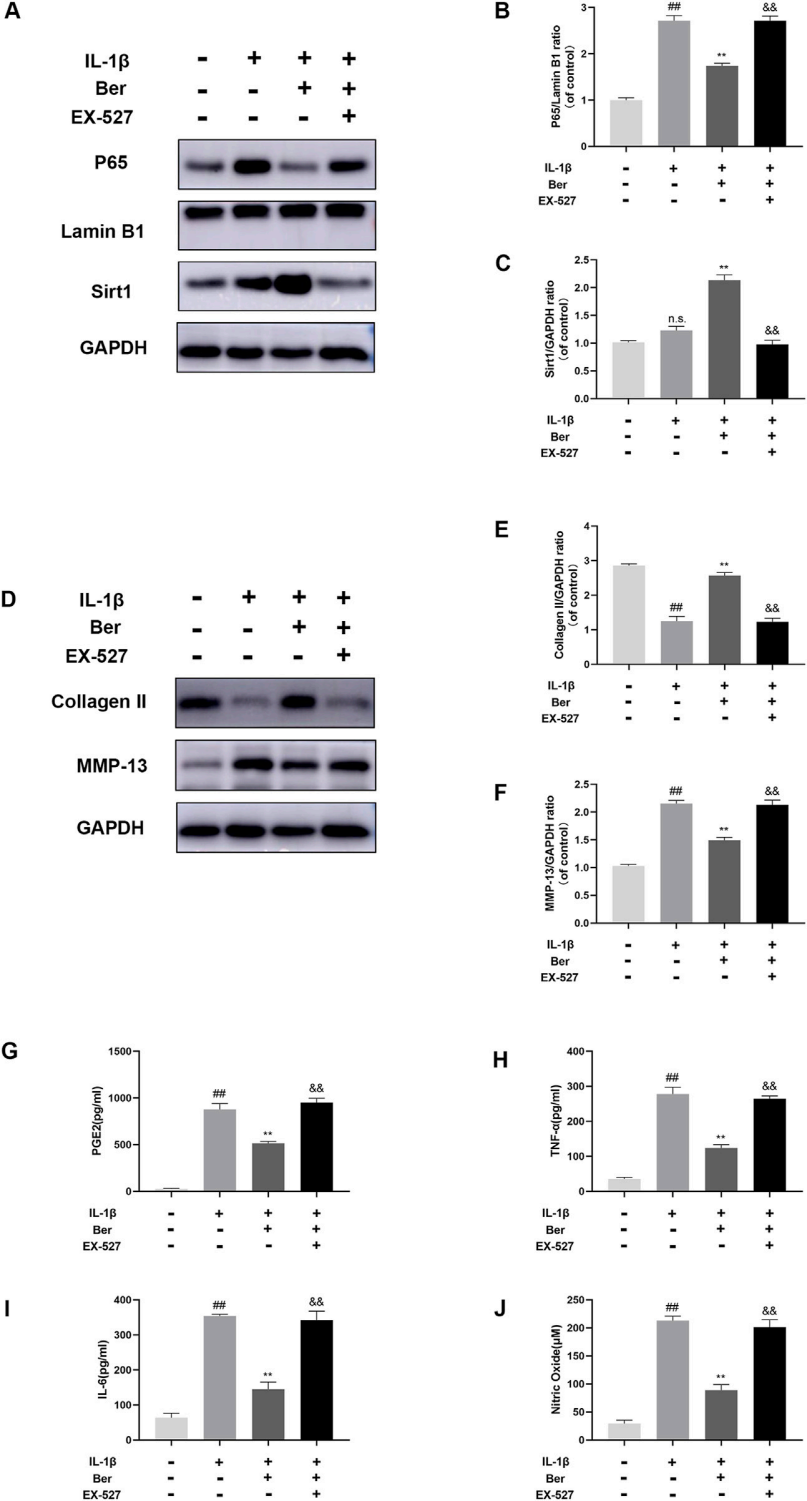
Ex-527 inhibits the effects of Ber in IL-1β-induced chondrocytes. **(A–C)** The level of P65 and Sirt1 in the chondrocytes. **(D–F)** The level of COL-Ⅱ and MMP-13 in the chondrocytes. **(G–J)** ELISA assay of IL-6 TNF-α, PGE2, AND NO in the chondrocytes. ##*p* ≤ 0.01 vs. control group; **p < 0.01 vs. IL-1β group, ^&&^ *p* < 0.01 vs. IL-1β+Ber group *n* = 5.

The potential mechanism underlying the activation of Sirt1 by Ber was investigated using EX-527 (an inhibitor of Sirt1). In the Western blotting analysis, EX-527 could significantly inhibits the expression of Sirt1, while the nuclear expression of p65 in chondrocytes was observed to increases (Figures 6A–C). In addition, EX-527 markedly eliminated the IL-1β-induced upregulation of COL-II expression in chondrocytes (Figures 6D–F). Furthermore, EX-527 eliminated the downregulation of MMP-13, NO, PGE2, TNF-α, and IL-6 in chondrocytes caused by treatment with Ber and IL-1β (Figures 6G–J).

### 3.7 Ber protected against OA development in a DMM model

**FIGURE 7:**
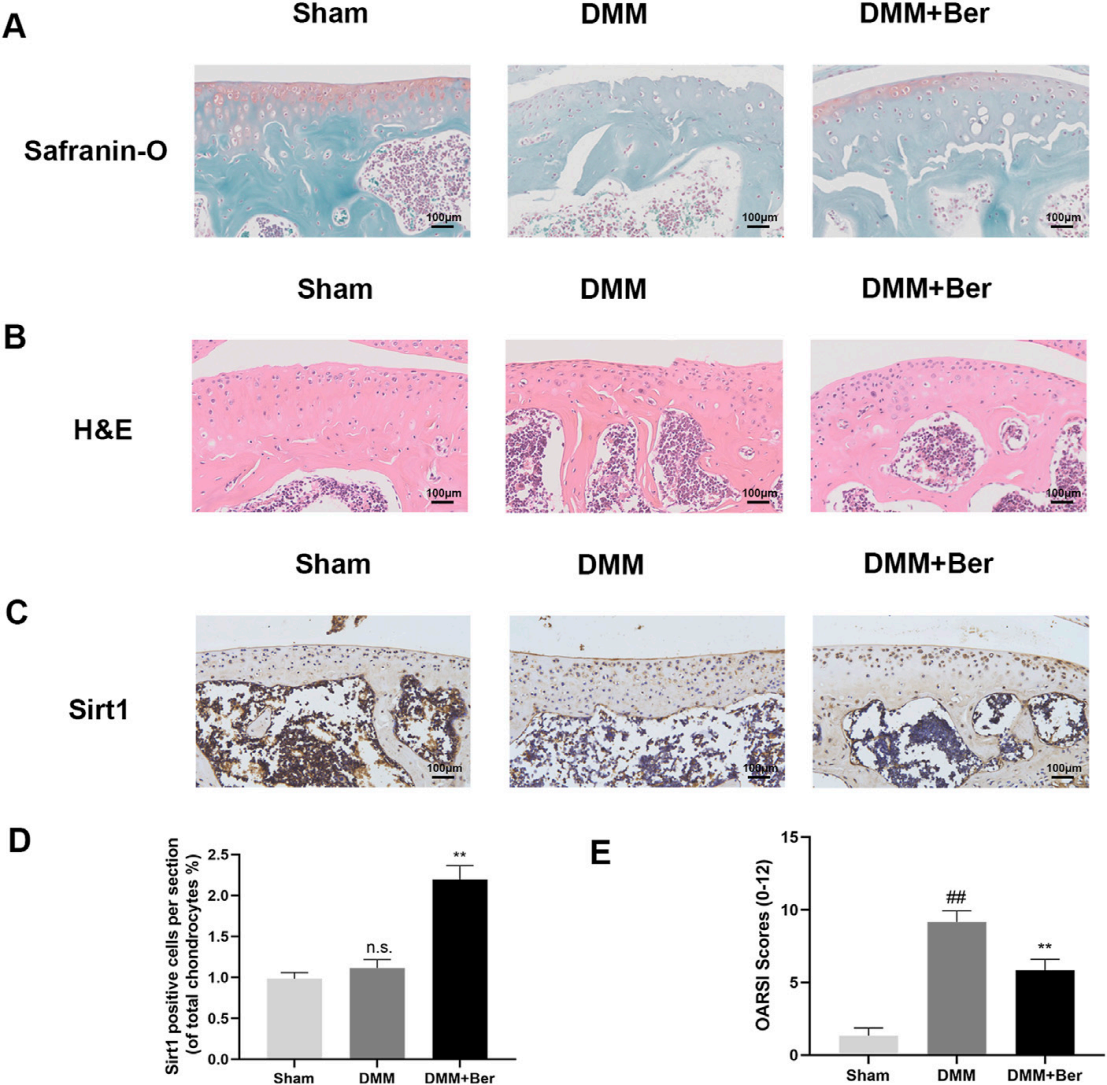
Ber inhibits the development of OA in the DMM model. **(A)** Safranin-O staining of cartilage. **(B)** H&E staining of cartilage. **(** **C, E** **)** Immunohistochemistry images of Sirt1 in the cartilage. **(D)** OARSI scores of cartilage. ##*p* ≤ 0.01 vs. Sham group; **p < 0.01 vs. DMM group, *n* = 6.

The effects of Ber on osteoarthritis (OA) were also investigated *in vivo*, The OA mouse model was established through DMM surgery. Afterward, these mice were administered with 20 mg/kg of Ber orally every day for the next 8 weeks. Safranin O staining was performed to assess the severity of OA in these mice. The results revealed that compared to the sham surgery group, the surgery group exhibited significant cartilage degradation, loss of extracellular matrix glycosaminoglycans, and cellular degeneration (Figure 7A). On the other hand, the group treated with Ber exhibited evident cartilage repair. H&E staining also revealed similar results and indicated that Ber had significantly improved osteoarthritis (Figure 7B). The subsequent immunohistochemical staining revealed a marked upregulation of Sirt 1 in the Ber group (Figures 7C,D). The changes in OARSI scores were also consistent with the results of H&E and Safranin O staining analyses, with the Ber group presenting lower than scores compared to the DMM group (Figure 7E). In summary, the above results provided evidence that Ber mitigated the progression of OA by limiting cartilage degradation, cartilage sclerosis, and the abnormal formation of bone spurs.

## 4 Discussion

OA is a common disease that poses a significant threat to human wellbeing and vitality. OA has emerged as the primary cause of disability among older individuals (Silverwood et al., 2015). Over 300 million people worldwide have their daily lives significantly impacted by the long-term suffering due to arthritis (Thysen et al., 2015). OA causes pain to the affected individual and also places a significant economic burden on the entire family of the patient. The current treatments for osteoarthritis are primarily focused on symptom relief; although the medications used often lead to serious side effects (Sun et al., 2017). The primary drugs used for relieving osteoarthritis are non-steroidal anti-inflammatory drugs, although the etiology of arthritis remains poorly understood to date (da Costa et al., 2017). Therefore, it is imperative to develop novel drugs targeting the specific molecular points in OA to achieve effective treatment of this disease.

Bergamottin is a common biologically active furanocoumarin, abundantly produced abundantly in grapefruits (Li et al., 2022; Zhang et al., 2022). Bergamottin has been drawing increasing interest recently owing to its anti-inflammatory properties. It is reported that Bergamottin alleviates LPS-induced acute lung injury by inducing SIRT1 and inhibiting NF-κB (An et al., 2021). Research also suggests that Bergamottin prevents LPS-induced endotoxic shock by modulating the NF-κB signaling pathway (Liu et al., 2022). Importantly, Bergamottin blocks the activation of NF-κB signaling (Mizuno et al., 2017). These findings suggest that Bergamottin might have anti-inflammatory effects on patients with osteoarthritis.

Substantial research indicates that the NF-κB pathway plays a crucial role in the regulation of inflammatory mediators during the development of osteoarthritis (Liacini et al., 2002). Under normal conditions, the subunit protein p65 of NF-κB is bound to IκBα in the cytoplasm (Ozawa et al., 2015). However, upon IL-1β induction, IκBα undergoes phosphorylation, followed by degradation, leading to the translocation of activated p65 from cell cytoplasm to the nucleus (W. Li et al., 2016). The translocation of p65 into the nucleus, it promotes the expression of the related inflammatory factors such as NO and IL-6 (Sasaki et al., 1998). The inflammatory factor iNOS elevates the expressions of NO and MMPs, leading to the degradation of the extracellular matrix (ECM) (Goldring and Goldring, 2004). The degradation of the extracellular matrix (ECM) and/or the upregulation of matrix-degrading enzymes in chondrocytes are crucial factors for cartilage destruction (Goldring et al., 1994). The present study demonstrated that Ber could reduce the levels of matrix metalloproteinase 13 (MMP13) and ADAMTS-5 proteins in LPS-induced chondrocytes while increasing the levels of type II collagen and Aggrecan.

SIRTUIN 1 is the mammalian ortholog of yeast SIR2 (silencing information regulator). It belongs to the sirtuin family and is associated with the modulation of cell survival and transcriptional silencing (Shi et al., 2021; Wu et al., 2021). SIRTUIN 1 interacts with the NF- κB RelA/p65 subunit and also deacetylates RelA/p65 at the lysine 31,011 residue to suppress transcription, thereby affecting the transcription of various apoptosis-related genes such as ARGs, BAX, Bcl-2, Cyto-c, or inflammation-related genes (IL-6 and IL-1β) and catabolic genes (MMP3, MMP13, and ADAMTSs) (Feng et al., 2019; Liu et al., 2021).

In the present study, Ber was demonstrated to activate Sirt1, which then inhibited the activation of the NF-κB pathways. In order to investigate the association between Sirt 1 and NF- κB activation, an inhibitor of Sirt 1 was employed to suppress the expression of Sirt 1 in chondrocytes. As depicted in Figure 6, Ber alleviated inflammation in IL-1β-induced chondrocytes through the activation of Sirt1/NF-κB, and EX-527 (an inhibitor of Sirt1) could significantly eliminate this effect of Ber. These results demonstrated that Ber increased the IL-1β-mediated ECM deterioration and inflammation within chondrocytes through the Sirt 1/NF-κB signal transduction pathway.

Collectively, the results of the present study demonstrated that Ber inhibited the IL-1β-induced inflammation via the Sirt1/NF-κB pathway. DMM, as a classic animal OA model, is used widely to simulate human OA diseases. In the present study, we found that the DMM group mice exhibited cartilage degradation, extracellular matrix reduction, and matrix proteoglycan loss. However, all of these phenomena were improved dramatically upon treatment with Ber.

**FIGURE 8:**
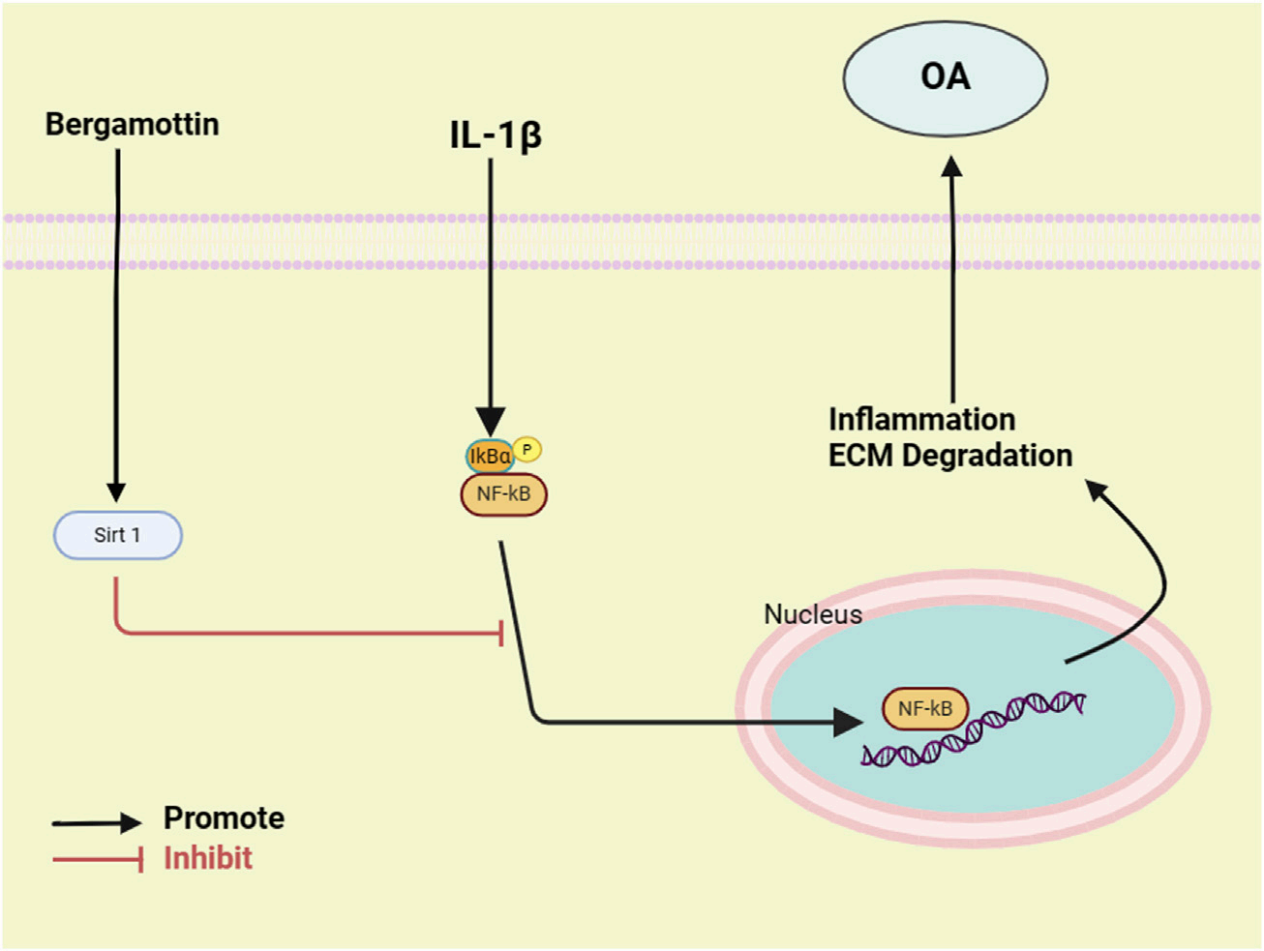
Diagram of the molecular mechanism of Ber involved in the progress of osteoarthritis.

Further, the findings of the present study suggested that Ber inhibits the activation of NF-κB by activating the Sirt1 pathway in chondrocytes, thereby significantly ameliorating the IL-1β-induced inflammation in these cells (Figure 8). Moreover, Ber also suppressed the degradation of ECM in the *in vivo* models. Therefore, the *in vivo* and *in vitro* data were consistent, demonstrating the value of Ber in the treatment of OA.

## 5 Conclusion

Ber inhibits the inflammatory conditions induced by IL-1β induction, including the reduced expressions of MMP-13, COX-2, INOS, and ADAMTS-5 in the OA chondrocytes. Moreover, Ber inhibits IL-1β-induced inflammation by activating the Sirt1/NF-κB pathway. Accordingly, the addition of Ber to the cell culture may protect against OA in an OA mouse model. Therefore, Ber may be used as an alternative therapeutic agent for the treatment of OA in the future.

## Data Availability

The original contributions presented in the study are included in the article/supplementary materials, further inquiries can be directed to the corresponding author.
